# Massachusetts Veterinary Association

**Published:** 1892-01

**Authors:** Austin Peters

**Affiliations:** Secretary


*Massachusetts Veterinary Association.*–The regular meeting of the Massachusetts Veterinary Association was held at 19 Boylsten Place, Boston, Wednesday evening, Nov. 25, 1891.

President L. H. Howard in the chair. Members present: Drs. Bryden, Blackwood, Becket, Burr, Emerson, Hadcock, Howard, Marshall, Winchester, and the Secretary. Honorary member, Dr. Stickney. Visitors Drs. W. W. Noyes and E. T. Harrington, and also Mr. Kendall, of Sterling, Mass.

Minutes of last meeting read and accepted.

Unfinished business: Amendment to Constitution providing that names of applicants for membership be laid on the table for one month before being acted upon. Ballot taken, resulted in nine votes cast, all in the affirmative.

Reports of Committees: Committee appointed to confer with a committee from Board of Control of the State Experiment Station, reported progress. Motion made by Dr. Hadcock, and seconded by Dr. Blackwood, that report be accepted. Carried. Executive Committe reported through the chairman, Dr. Marshall, favorably upon the credentials of Dr. E. T. Harrington.

Admission of members: Ballot taken on Dr. E. T. Harrington's name, resulted in eleven votes cast, all in the affirmative.

Applications for membership: Secretary directed to write personal letters to Dr. J. M. Parker, of East Rindge, N. H., and to Dr. W. A. Hitchcock, of Malden, informing them of the conditions of membership in this Association.

New business: Moved by Dr. Winchester, and seconded by Dr. Hadcock, that the Secretary have a number of copies of the constitution printed, together with a number of circulars and forms and mail them at his discretion to members of the veterinary profession. Carried.

Papers and discussions; Paper upon "Springhalt" by Dr. Williamson Bryden, being a report upon his treatment of the old black gelding kept for experimental purposes for this Association, by Mr. Andrew Ward, of the N. Ward Co's Wharf, Boston, and killed for autopsy July 27th, 1891, together with the essayist's views upon this affection. *Vide fol. 12.*

A spirited discussion followed, in which the members and visitors took part, Most of those present did not agree with the essayist in all of his views, especially the idea that in springhalt, spavin, and the like, the first change is in the foot, and that the other troubles are consequent upon it, believing, on the contrary, that the alteration in the shape of the foot is the result of the disease above. Nearly all who had seen the horse before and after treatment by Dr. Bryden admitted that there was an improvement in his condition at the end of the course.

The Secretary read a note from Dr. H. H. Donaldson, of Clark University, in which he reported that he had nearly finished the misroscopic examination of the horse's spinal cord, from the lumbo-sacral region, without finding any pathological changes. If before finishing the examination any changes were found, it would be reported to the Association.

Dr. Howard brought the bones of the near hock, the cuboid scaphoid, large and small cuneiform were anchylosed together and to the metatarsal bone. There also appeared to be a little ulceration upon the trochlea of the astragalus, but the bones had been soaked out in rather a strong pickle rendering this uncertain.

Moved by Dr. Marshall, and seconded by Dr. Becket, that the essayist be given a vote of thanks. Carried.

Meeting then adjourned.

AUSTIN PETERS, *Secretary.*

